# Chronic disease (CD) during transition from child to adult.Psychopathological consequences and coping strategies

**DOI:** 10.1192/j.eurpsy.2023.1565

**Published:** 2023-07-19

**Authors:** S. Rubio Corgo, M. Arrieta Pey, A. M. Matas Ochoa, M. I. Duran Cristobal, E. Perez Vicente, A. Delgado Campos, C. Diaz Gordillo, A. C. Castro Ibañez, A. Alvarez Astorga, P. Alcindor Huelva

**Affiliations:** 1 Psiquiatría, Hospital Infanta Leonor, Madrid, Spain

## Abstract

**Introduction:**

CD is characterized by at least three features: its duration is prolonged, it does not resolve spontaneously and it is rarely completely cured. Approximately 10-15% of young people have CD. Adolescents with CD often have emotional and behavioral problems.

**Objectives:**

To assess risk factors, derived psychiatric pathologies and coping strategies for a CD diagnosis in adolescence.

**Methods:**

An extensive literature review was carried out on the subject in question, extracting information mainly from scientific articles, manuals and books.

**Results:**

The main risk factors are those related with the CD in question, physical sequelae, the need for long-term hospital admissions or the use of drugs whose side effects include affective or behavioral symptoms; those related to the personality traits of the affected child or adolescent. In addition, as far as the family is concerned, the presence of a low level of education, lack of support or communication, as well as the presence of psychiatric disorders or serious medical conditions in parents. Among the most frequent psychiatric disorders associated with CD are affective and anxiety disorders, adaptive disorders, somatoform disorders, eating disorders and behavioral disorders. Whatever the CD is, it generates high levels of stress and uncertainty in the patient and family, which must be dealt together from a flexible perspective, allowing child or adolescent to adapt to the changes, reorganize and facing them with adaptive patterns of behavior. For this, it will be essential to have adequate social and family support with relational style based on communication, trust and acceptance.

**Conclusions:**

In general, both adolescents with CD and their families have an adequate capacity to adapt to the repercussions and effects derived from the disease. Nevertheless, in case of possible emotional difficulties that may appear, a comprehensive and individualized approach to these adolescents and their families is necessary to provide them resources and coping strategies in different areas and contexts in which the disease debuts.The comprehensive therapeutic approach will consist of interventions at the individual and family level. Among the main objectives of these interventions are to achieve acceptance and adaptation to CD provinding adequate psychosocial support to enable them to cope with CD in the best possible way and to detect and address the emotional implications, even coexisting psychopathology.

**Disclosure of Interest:**

None Declared

